# Primary neuroendocrine breast cancer—an unusual occurrence

**DOI:** 10.3332/ecancer.2023.1520

**Published:** 2023-03-16

**Authors:** Anjana Vinod, Ishika Mahajan, Aruni Ghose, Saraswathy Sreeram

**Affiliations:** 1 Department of Medical Oncology, Kasturba Medical College, Mangalore Manipal Academy of Higher Education, Manipal 575001, India; 2 Apollo Cancer Centre, Chennai 600035, India; 3 Department of Medical Oncology, Barts Cancer Centre, St. Bartholomew’s Hospital, Barts Health NHS Trust, London EC1B 7BE, UK; 4 Department of Medical Oncology, Mount Vernon Cancer Centre, East and North Hertfordshire NHS Trust, London HA6 2RN, UK; 5 Research and Innovation, Medical Oncology, Medway NHS Foundation Trust, Kent ME7 5NY, UK; 6 Division of Research, Academics and Cancer Control, Saroj Gupta Cancer Centre and Research Institute, Kolkata 700063, India; 7 Department of Pathology, Kasturba Medical College, Mangalore Manipal Academy of Higher Education, Manipal 575001, India

**Keywords:** neuroendocrine, breast cancer, NEBC, primary, synaptophysin, chromogranin, case report

## Abstract

Primary breast neuroendocrine neoplasms (BNENs) are a rare form of breast cancer, accounting for less than 0.1% of all breast malignancies. These neoplasms have a similar clinical presentation as conventional breast carcinomas, differing mainly in their histopathology and expression of neuroendocrine (NE) markers, chromogranin and synaptophysin. Owing to their rarity, current knowledge of these tumours comes mainly from corroborative case reports or retrospective case series. There is hence a lack of randomised data on the treatment of these entities and current protocol suggests similar treatment as that of conventional breast carcinomas. We report the case of a 48-year-old with a breast mass, which on further work-up was diagnosed as locally advanced carcinoma breast, that required a mastectomy and axillary node dissection on the same side and revealed NE differentiation on histopathological examination. Hence, immunohistochemical staining was indicated which confirmed NE differentiation. We discuss the current knowledge on incidence, demographics, diagnosis, histopathological and staining characteristics, prognostic factors and treatment modalities of BNENs.

## Introduction

Primary breast neuroendocrine neoplasms (BNENs) constitute an under-recognised subtype of breast malignancies. This entity accounts for less than 0.1% of all cases of carcinoma breast and under 1% of primary neuroendocrine neoplasms (NENs) [1]. A characteristically diverse group of tumours originating from neuroendocrine (NE) cells, NENs have the propensity to arise in a plethora of locations, notably in the gastrointestinal tract, central nervous system, respiratory system, skin and skin [2].

The updated WHO Classification of Tumours of the Breast, in 2019, defined BNEN as a tumour with greater than 90% of cells showing NE differentiation, that is, expressing NE markers — chromogranin and synaptophysin (SYN) [3]. First described by Cubilla and Woodruff [4] in a series of eight cases, these cases have been rarely encountered since. These cases are heterogeneous in terms of degrees of differentiation and histological characteristics. They do not show a unique natural clinical history, making it difficult to raise a degree of suspicion for them at presentation. There is a dearth of data on building this natural history due to the rarity of cases, thus not much is known in terms of specific therapies, effectiveness and prognosis [5].

The present case report outlines a unique case of a primary BNENs.

## Case presentation

A 48-year-old nulliparous lady without comorbidities presented with a 3-month history of a lump in the lower outer quadrant of her left breast. On examination, a 2 × 2 cm, irregular, hard lump fixed to the surrounding breast tissue was palpated. Multiple, non-tender enlarged level 1 lymph nodes were also felt in the left axilla.

Mammography showed an irregular, infiltrating, Breast Imaging-Reporting and Data System (BIRADS) -V lesion in the posterior aspect of the lower outer quadrant measuring 2 × 2 × 1 cm. Significant left axillary lymphadenopathy was also noted.

Fine needle aspiration cytology of a level 1 axillary lymph node revealed metastatic breast cancer. Ultrasound-guided tru-cut biopsy of the breast lump yielded nests of cells with nuclear moulding and coarse chromatin. A provisional diagnosis of carcinoma with NE differentiation was made.

A chest X-ray showed normal findings and an ultrasound study of the abdomen and pelvis revealed post-hysterectomy status with no other sonological abnormalities.

She underwent a left modified radical mastectomy with axillary node clearance, following the workup.

**Figure 1.**
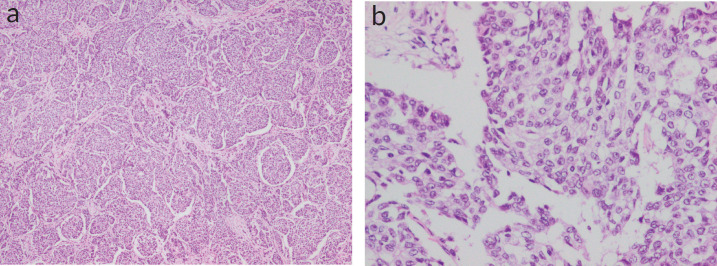
(a and b): Histopathological examination showing densely cellular nests and trabeculae with rounded margins with delicate fibrovascular stroma separating them. Neoplastic cells were larger and polygonal, with eosinophilic, granular cytoplasm and coarse and fine nuclear chromatin. (Figure 1a — 4× magnification; 1b — 20× magnification).

On gross examination, a 3 × 2.5 × 1 cm, grey-white, well-circumscribed, unifocal, firm mass was noted. Histologically, the tumour was composed of densely cellular nests and trabeculae. The cells were arranged as solid sheets with rounded margins with delicate fibrovascular stroma separating them. Neoplastic cells were larger and polygonal, with eosinophilic, granular cytoplasm and coarse and fine nuclear chromatin (Figure 1a and b).

**Figure 2.**
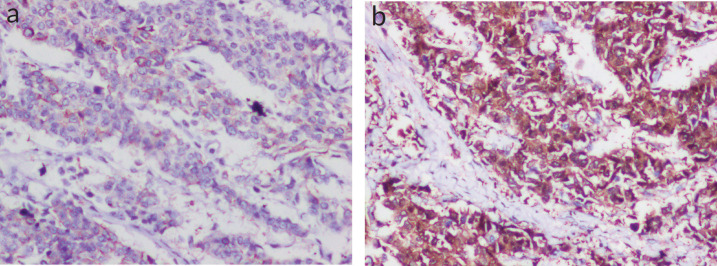
(a and b): IHC panel for NE markers (Figure 3a — NSE-positive status; 3b — SYN-positive status).

**Figure 3.**
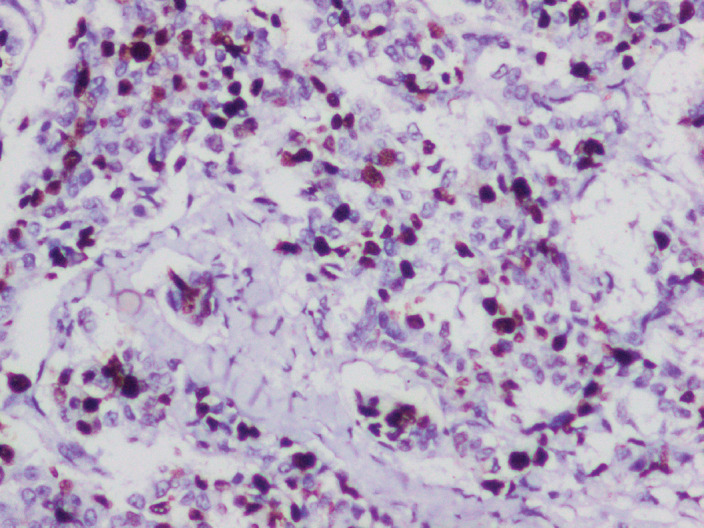
IHC for ki-67 proliferation index showing 10%.

Lympho-vascular invasion was present. Four out of the ten dissected lymph nodes showed metastatic deposits, but there was no extra nodal extension. The tumour tubule formation score was 3 and nuclear pleomorphism was scored at 1. The mitotic activity was 2, and the tumour was given a Nottingham Grade 2. No component of ductal carcinoma *in situ* (DCIS) was found. Electron microscopy revealed dense core secretory granules. On Immunohistochemistry (IHC), the tumour was hormone receptor- oestrogen and progesterone (ER, PR) positive and negative for HER2/neu (a non-hormone IHC marker for breast cancer that guides treatement options). More than 90% of the cells were strongly positive for neuron-specific enolase (NSE) and SYN (Figure 2a and b). Chromogranin was negative. Ki-67 proliferation index was 10% (Figure 3). A final diagnosis of invasive carcinoma with NE features was made. The staging was pT2N2a.

She received standard-of-care therapy with four cycles of Adriamycin (60 mg/m^2)^ and Cyclophosphamide (600 mg/m^2^) followed by four cycles of single-agent docetaxel (75 mg/m^2^). The chemotherapy was followed by adjuvant radiation. 50 Gy of three-dimensional conformational radiation therapy with radical intent was given in 25 fractions without concurrent chemotherapy.

Post-menopausal status was confirmed with inhibin-B levels and a Pap smear. Ultrasound abdomen and pelvis was unremarkable. Therapy with letrozole 2.5 mg was commenced.

After 12 months of letrozole therapy and regular 2 monthly follow-ups, a right breast mammogram was done. It showed BIRADS I status with no disease spread to the right breast. She remained clinically asymptomatic. Letrozole has since been continued.

She is on surveillance at present. Current disease-free interval is 2 years.

## Discussion

BNENs, as discussed above, are a rare subtype of NENs, which the WHO did not describe until 2003. Presently, they are found to account for about 0.1%–5% of all breast cancers. A mini review done by Gallo *et al* [5] revealed under 200 cases to be reported in the literature. This further impresses their rarity and the importance of reporting individual unique cases such as this one, to shed light on the nature and incidence of the disease.

Wang *et al* [6] reported that BNENs comprised <0.1% of all invasive breast carcinomas and patients tend to be older women, frequently postmenopausal, most presenting in the sixth decade of life, with larger tumours or positive regional lymph node status at the time of diagnosis as compared to invasive ductal carcinoma, not otherwise specified (IDC-NOS). In our case, the patient does not completely fit into this demographic, but she did present with lymph node positivity and postmenopausal status.

Owing to the rarity of incidence of these tumours, literature on their specific clinical features is limited. Primary BNEN presents with similar clinical features as other breast cancer types, frequently as a palpable painless breast lump with or without enlarged regional lymph nodes. There may be associated skin changes [7]. This presenting complaint has also been noted in our case.

Imaging findings in these neoplasms are indistinguishable from other mammary malignancies. Mammography findings are usually descriptive of hyperdense, irregular, spiculated and micro lobulated mass lesions. Breast ultrasound generally picks up these neoplasms as hypoechoic masses, with ill-defined margins [8]. The aforesaid were consistent in our patient. Magnetic resonance imaging suggests irregular mass with poorly defined margins and washout kinetics [9].

The histological origin of BNEN is still unclear but the latest data postulates two theories. Some researchers propose that these tumours arise due to differentiation in breast cancer cells as NE cells have not been found in breast tissues, while another school of thought suggests NE cell proliferation in breast parenchyma in isolated clusters may be related to some precancerous states [10].

On histopathology, neuroendocrine tumours (NETs) show similar round or spindle-shaped cells with palisading of the nucleus, abundant eosinophilic and fine granular cytoplasm, and nuclei with ‘salt and pepper chromatin’. Tumour cells form islands, nests and trabeculae which are surrounded by a delicate fibrovascular stroma. According to the WHO classification of tumours 2019, essential criteria for BNENs include histological and immunohistochemical features of NE differentiation and co-existing DCIS is described as a desirable criterion [3]. In the present case, the patient presented at stage T2 with histology consistent with NE features such as solid sheets of cells with rounded margins, separated by fibrovascular stroma and granular, eosinophilic cytoplasm and coarse chromatin but DCIS was found to be absent. This is a key point to note as this is a desirable criterion in the classification, which is not present, while the essential criteria to diagnose BNEN have been fulfilled in this patient.

WHO describes BNENs as tumours with >90% of cells showing histological and IHC features of NE differentiation, mainly the expression of NE markers such as SYN and chromogranin A (CGA). CD56 and NSE may also be positive but are not as specific [3]. In addition, there may be the appearance of neurosecretory granules. NEBC, as described in literature, is by nature hormone receptor positive and HER2/neu negative. This type is called luminal type A breast cancer. Luminal B type may also be present [11]. Krawczyk *et al* [12] found the most common immunohistochemical subtype to be ER, PR+ and HER2/neu negative. 93% of samples were positive for SYN and 48% for CGA [12]. Previous studies have found an overall expression rate of 30%–72% for CGA and 74%–100% for SYN. It is also worth noting that BNEN may not always show CGA/SYN-positive staining [13]. In our patient, resected breast tissue showed cells with typical NE features and was positive for oestrogen and progesterone receptors (ER+, PR+) and negative for HER2/neu. Tumour cells (>90%) were strongly positive for SYN and positive for NSE; however, chromogranin was negative. Rovera *et al* [14] noted the Ki-67 proliferation index for BNENs to be on the lower side (14%), which might indicate a better prognosis for these tumours. In our patient, the Ki-67 proliferation index was 10%, which corroborates with the existing literature.

As clinical characteristics and imaging features of BNEN are non-specific, histological features and immunohistochemical staining of tissues remain the cornerstone of diagnosis.

As per 2019 WHO classification, well-differentiated BNENs were described as NETs which are considered low to intermediate-grade tumours in the current classification. Poorly differentiated BNENs, earlier described as NECs (neuroendocrine carcinoma) are considered high-grade tumours which include small-cell NEC and large-cell NEC [3]. BNEN patients are found to usually present at a higher clinical stage and higher histological grade than IMC-NOS [6].

The differential diagnosis for these primary carcinomas is essentially metastasis from NECs at other sites. Presence of DCIS and the absence of tumours in other organs in radiology help in clinching the diagnosis of primary NEC of the breast [10]. In the current case, DCIS was not found, however, there were no symptoms or radiological findings suggesting metastasis from other organs.

In terms of the prognosis of BNENs, the current literature is rather ambiguous with different investigators presenting conflicting reports. This ambiguity can also be attributed to the comparative smaller incidence of these tumours and the lack of larger studies on them.

Previously, based on small studies, BNENs were thought to have a prognosis similar [15] or even better [16] compared to IDC-NOS. However, more recent studies by Wang *et al* [6] and Yang *et al* [17] suggest BNENs have shorter disease-free survival (DFS), higher chances of recurrence and worse overall survival (OS) than IDC. Another study has suggested that BNENs have shorter DFS, but OS is similar when compared to IDC-NOS [18]. Cloyd *et al* [19] have shown that DFS and OS are worse with BNEN with small cell histological subtype compared to well-differentiated BNENs. This finding is supported by Lavigne *et al* [18] where the 7 poorly differentiated NECs out of 47 BNENs analysed in the study, had worse OS when compared to other groups. It was also found that BNENs expressing higher levels of NE biomarkers were associated with less aggressive clinical parameters such as lower stage, lower histological grade and lesser lymph node metastasis [20]. In the existing literature, histological grade, disease stage, number of lymph node metastases and hormonal receptors status have been found to be prognostic factors affecting survival. In most studies, hormone receptor-negative status, larger tumour size (>20 mm), Ki-67 index > 14%, higher tumour stage and lack of surgical treatment have shown poorer prognosis [21].

Presently, there is limited information to recommend a specific treatment protocol for invasive breast carcinomas with NE differentiation and current guidelines suggest treatment same as that of conventional breast cancer (IDC-NOS).

Surgery remains the mainstay of treatment for early-stage BNEN. The type of surgery is decided upon by criteria similar to that of IDC-NOS. Of these, the location of the tumour and its size determines the surgical method. There is no sufficient evidence to date showing the most efficient chemotherapy regimen for these cancers. Generally, chemotherapy used in patients depends on the histological features of the patient’s tumour. Regimens containing platinum-etoposide, usually indicated for small-cell lung cancer, can be used in poorly differentiated small-cell BNENs [22], while anthracyclines and/or taxanes have been indicated in other BNENs [23]. There is a lack of sufficient evidence at present to suggest whether BNENs should be treated with neoadjuvant chemotherapy. A case by Wei *et al* [23] in 2015 reported a remarkable response to neoadjuvant chemotherapy. The current consensus is that adjuvant systemic therapy should be decided on an individual basis depending on the patient’s condition, with the same principles used for IDC-NOS [7]. Adjuvant endocrine therapy can be used in patients with hormone receptor positive BNENs. Anti-Her2 agents are suitable in Her2/neu positive status, which are mostly sporadic cases of BNEN [24]. Somatostatin analogues show antiproliferative effects and inhibitory effects on hormonal secretion in small intestinal NECs [25]. Studies studying the expression of SSTR2 and SSTR5 receptors in BNENs are sparse. Notably, Terlević *et al* [26] stated that 71% of BNENs express SSTR2A and SSTR5 receptors, making them potential therapeutic targets. Peptide receptor radionuclide therapy may also be useful in metastatic BNENs [27].

Our patient was surgically treated with a modified radical mastectomy of the affected breast with standard adjuvant chemotherapy indicated for Her2/neu negative breast carcinomas. Afterwards, she was started on adjuvant endocrine therapy and has since been in clinical remission. Treatment given was independent of NE features and according to principles of treatment for IDC-NOS, as indicated in the existing literature.

## Conclusion

There is an unmet need for further studies into these rare tumours for further understanding of these unusual clinical entities and for developing new treatment interventions. This case strengthens the importance of histopathology and early identification of NE features in patients with invasive breast cancer, which will help in early treatment and improved outcomes.

## Conflicts of interest

The authors have no conflicts of interest to declare.

## Funding

There was no funding received for publishing this case report.

## References

[1] Collado-Mesa F, Net JM, Klevos GA (2017). Primary neuroendocrine carcinoma of the breast: report of 2 cases and literature review. Radiol Case Rep.

[2] Oronsky B, Ma PC, Morgensztern D (2017). Nothing but NET: a review of neuroendocrine tumors and carcinomas. Neoplasia.

[3] WHO (2019). Classification of Tumours. Breast Tumours.

[4] Cubilla AL, Woodruff JM (1977). Primary carcinoid tumor of the breast. A report of 8 patients. Am J Surg Pathol.

[5] Gallo M, Campione S, Di Vito V (2021). Primary neuroendocrine neoplasms of the breast: still open issues. Front Endocrinol.

[6] Wang J, Wei B, Albarracin CT (2014). Invasive neuroendocrine carcinoma of the breast: a population-based study from the surveillance, epidemiology and end results (SEER) database. BMC Cancer.

[7] Inno A, Bogina G, Turazza M (2016). Neuroendocrine carcinoma of the breast: current evidence and future perspectives. Oncologist.

[8] Ozdemir O, Zengel B, Yildiz Y (2021). Neuroendocrine differentiated breast cancer cases: a retrospective analysis and literature review. Sisli Etfal Hastan Tip Bul.

[9] Park Y, Wu Y, Wei W (2014). Primary neuroendocrine carcinoma of the breast: clinical, imaging, and histologic features. AJR Am J Roentgenol.

[10] Sun H, Dai S, Xu J (2022). Primary neuroendocrine tumor of the breast: current understanding and future perspectives. Front Oncol.

[11] Ozaki Y, Miura S, Oki R (2021). Neuroendocrine neoplasms of the breast: the latest WHO classification and review of the literature. Cancers (Basel).

[12] Krawczyk N, Röwer R, Anlauf M (2021). Invasive breast carcinoma with neuroendocrine differentiation: a single-center analysis of clinical features and prognosis. Geburtshilfe Frauenheilkd.

[13] Tsang JY, Tse GM (2021). Breast cancer with neuroendocrine differentiation: an update based on the latest WHO classification. Mod Pathol.

[14] Rovera F, Lavazza M, La Rosa S (2013). Neuroendocrine breast cancer: retrospective analysis of 96 patients and review of literature. Int J Surg.

[15] Miremadi A, Pinder SE, Lee AH (2002). Neuroendocrine differentiation and prognosis in breast adenocarcinoma. Histopathology.

[16] Rovera F, Masciocchi P, Coglitore A (2008). Neuroendocrine carcinomas of the breast. Int J Surg.

[17] Yang L, Roy M, Lin H (2021). Validation of prognostic significance of the proposed uniform classification framework in neuroendocrine neoplasms of the breast. Breast Cancer Res Treat.

[18] Lavigne M, Menet E, Tille J (2018). Comprehensive clinical and molecular analyses of neuroendocrine carcinomas of the breast. Modern Pathol.

[19] Cloyd JM, Yang RL, Allison KH (2014). Impact of histological subtype on long-term outcomes of neuroendocrine carcinoma of the breast. Breast Cancer Res Treat.

[20] Lai B, Tsang J, Poon I (2020). The clinical significance of neuroendocrine features in invasive breast carcinomas. Oncologist.

[21] Patel G, Bipte S (2020). Updates in primary neuroendocrine breast carcinoma - a case report and review of literature. J Cancer Res Ther.

[22] Atchison L, Hardy T, Mancl T (2021). Locally advanced primary small cell carcinoma of the breast: a case report and review of current evidence. Case Rep Oncol.

[23] Wei X, Chen C, Xi D (2015). A case of primary neuroendocrine breast carcinoma that responded to neo-adjuvant chemotherapy. Front Med.

[24] Marijanović I, Kraljević M, Buhovac T (2020). Rare human epidermal growth factor receptor 2 (HER-2)-positive neuroendocrine carcinoma of the breast: a case report with 9-year follow-up. Am J Case Rep.

[25] Bongiovanni A, Recine F, Foca F (2018). Metastatic neuroendocrine neoplasia treatments in patients over 70 years of age. Endocr Connect.

[26] Terlević R, Perić Balja M, Tomas D (2019). Somatostatin receptor SSTR2A and SSTR5 expression in neuroendocrine breast cancer. Ann Diagn Pathol [Internet].

[27] Savelli G, Zaniboni A, Bertagna F (2012). Peptide receptor radionuclide therapy (PRRT) in a patient affected by metastatic breast cancer with neuroendocrine differentiation. Breast Care (Basel).

